# Ammonium diphenyl­phosphinate monohydrate

**DOI:** 10.1107/S1600536808012907

**Published:** 2008-05-07

**Authors:** Dongyang Li, Juan Chen, Jianping Guo

**Affiliations:** a Institute of Applied Chemistry, Shanxi University, Taiyuan 030006, People’s Republic of China; b Department of Chemistry, Taiyuan Teachers’ College, Taiyuan 030031, People’s Republic of China

## Abstract

In the title salt, NH_4_^+^·C_12_H_10_O_2_P^−^·H_2_O, the ion pair and water mol­ecule inter­act through hydrogen bonds to form a layer structure.

## Related literature

For other ammonium diphenyl­phosphinates, see: Guo *et al.* (2005 ▶); Dorn *et al.* (2001 ▶).
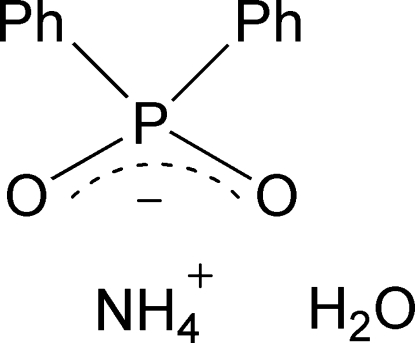


## Experimental

### 

#### Crystal data

NH_4_^+^·C_12_H_10_O_2_P^−^·H_2_O
*M* *_r_* = 253.23
Monoclinic, 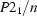

*a* = 15.027 (2) Å
*b* = 6.4594 (9) Å
*c* = 15.484 (2) Å
β = 117.394 (2)°
*V* = 1334.4 (3) Å^3^
*Z* = 4
Mo *K*α radiation
μ = 0.20 mm^−1^
*T* = 293 (2) K
0.20 × 0.20 × 0.15 mm

#### Data collection

Bruker SMART diffractometer
Absorption correction: multi-scan (*SADABS*; Sheldrick, 1996 ▶) *T*_min_ = 0.798, *T*_max_ = 0.970
6237 measured reflections
2341 independent reflections
2208 reflections with *I* > 2σ(*I*)
*R*_int_ = 0.021

#### Refinement

*R*[*F* ^2^ > 2σ(*F* ^2^)] = 0.064
*wR*(*F* ^2^) = 0.139
*S* = 1.22
2341 reflections
154 parameters
H-atom parameters constrained
Δρ_max_ = 0.35 e Å^−3^
Δρ_min_ = −0.23 e Å^−3^

### 

Data collection: *SMART* (Bruker, 2000 ▶); cell refinement: *SAINT* (Bruker, 2000 ▶); data reduction: *SAINT*; program(s) used to solve structure: *SHELXS97* (Sheldrick, 2008 ▶); program(s) used to refine structure: *SHELXL97* (Sheldrick, 2008 ▶); molecular graphics: *SHELXTL/PC* (Sheldrick, 2008 ▶); software used to prepare material for publication: *SHELXTL/PC*.

## Supplementary Material

Crystal structure: contains datablocks I, global. DOI: 10.1107/S1600536808012907/ng2451sup1.cif

Structure factors: contains datablocks I. DOI: 10.1107/S1600536808012907/ng2451Isup2.hkl

Additional supplementary materials:  crystallographic information; 3D view; checkCIF report

## Figures and Tables

**Table 1:** Hydrogen-bond geometry (Å, °)

*D*—H⋯*A*	*D*—H	H⋯*A*	*D*⋯*A*	*D*—H⋯*A*
N1—H1*B*⋯O2	0.88	1.94	2.814 (3)	170
N1—H2*B*⋯O1^i^	0.88	1.87	2.752 (3)	176
N1—H3*B*⋯O2^ii^	0.88	1.91	2.764 (3)	164
N1—H4*B*⋯O3^iii^	0.88	1.94	2.816 (4)	172
O3—H3*C*⋯O1^i^	0.86	1.89	2.723 (4)	164

Symmetry codes: (i) 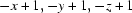; (ii) 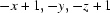; (iii) 
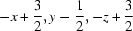
.

**Fig. 1.**
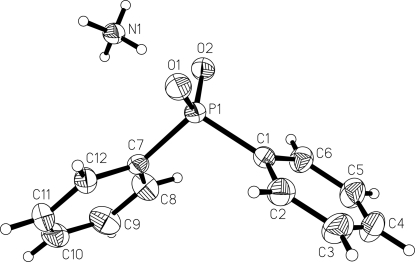
The molecular structure, showing the atom-numbering scheme. Displacement ellipsoids were drawn at the 30% probability level. The water molecule was omitted.

**Fig. 2.**
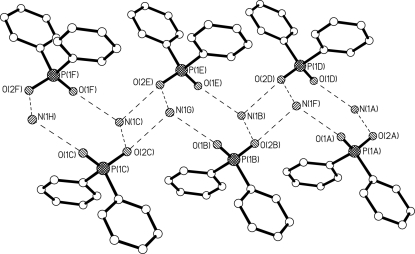
The infinite chain. The waters and all of H atoms were omitted.

## supplementary crystallographic information

### Comment

The title compound is a by-product when synthesizing 3-Cyanophenyl-amidinium diphenylphosphinate. Within the OPO fragment of the diphenylphosphinate anion, the P—O distances are 1.495 (2) and 1.503 (2) Å. The similar values was reported in the structure of arylamidinium diphenylphosphinate (Guo *et al.*, 2005). The P—O distances indicate that the charge of the diphenylphosphinate anion [Ph_2_PO_2_]^-^ is delocalized over the O—P—O framework. There are two types of hydrogen bond, namely P—O···H—N and P—O···H—O. The O—N distances are in the range of 2.752 (3)–2.816 (4) Å. The O—O distance is 2.723 (4) Å.

### Experimental

1,3-Dicyanobenzene (0.38 g, 3 mmol) and LiN(SiMe_3_)_2_ (1.0 g, 6 mmol) were dissolveded in THF (30 cm^3^) at 0°C. The resultant yellow solution was warmed to room temperature and stirred for an additional 2 h before cooling down to -78°C. Chlorodiphenylphosphine (1.1 cm^3^, 6 mmol) was then slowly added to the reaction mixture which was stirred at -78°C for an hour before warming up to room temperature and allowed to react overnight. Solvent was then removed in vacuum. The residue was extracted with dichloromethane and the solution was filtered. The solvent of the filtrate was removed in vacuum to give a dark red oilyproduct. The product was dissolved in acetonitrile (30 cm^3^) and 30% hydrogen peroxide (0.68 cm^3^, 6 mmol) was added in air. After stirring for 24 h at room temperature, the reaction mixture was filtered. The colorless crystals of compound 3-Cyanophenyl-amidinium diphenylphosphinate were produced first; then colorless crystals of the title compound were obtained. Yield: 0.50 g, 2.1 mmol, m.p. 185–187 °C. ^1^H NMR (300 MHz, [D_6_]DMSO): d = 7.27 (m, 6H, Ar),7.61–7.64 (m, 4H, Ar). ^13^CNMR (75 MHz, [D_6_]DMSO): δ = 130.7, 130.9,132.7, 134.2, 144.1. ^31^P NMR (121.5 MHz, [D_6_]DMSO): δ = 13.3. IR (cm^-1^,in KBr): 3611*m*, 3071 b s, 3009 b s, 2833 b s, 1638*m*, 1483 s, 1400*m*, 1163vs, 1128vs, 1068*m*, 1040vs, 1020 s, 962*m*, 725vs, 694 s, 565vs.

### Refinement

The ammonium and water H atoms were found by using fourier difference map and constrained to their related atoms, with N—H distances in the range 0.88 Å and *U*_iso_(H) = 1.2*U*_eq_(N), O—H distances in the range 0.86 Å and *U*_iso_(H) = 1.2*U*_eq_(O). The phenyl H atoms were placed in geometrically idealized positions and constrained to ride on their parent atoms, with C—H distances in the range 0.93 Å and *U*_iso_(H) = 1.2*U*_eq_(C).

### Crystal data

**Table:**

NH_4_^+^·C_12_H_10_O_2_P^–^·H_2_O	*F*_000_ = 536
*M**_r_* = 253.23	*D*_x_ = 1.260 Mg m^−^^3^
Monoclinic, *P*2_1_/*n*	Mo *K*α radiation λ = 0.71073 Å
Hall symbol: -P 2yn	Cell parameters from 3307 reflections
*a* = 15.027 (2) Å	θ = 2.6–27.5º
*b* = 6.4594 (9) Å	µ = 0.20 mm^−^^1^
*c* = 15.484 (2) Å	*T* = 293 (2) K
β = 117.394 (2)º	Block, colorless
*V* = 1334.4 (3) Å^3^	0.20 × 0.20 × 0.15 mm
*Z* = 4	

### Data collection

**Table:**

Bruker SMART diffractometer	2341 independent reflections
Radiation source: fine-focus sealed tube	2208 reflections with *I* > 2σ(*I*)
Monochromator: graphite	*R*_int_ = 0.021
*T* = 293(2) K	θ_max_ = 25.0º
ω scans	θ_min_ = 2.6º
Absorption correction: multi-scan(SADABS; Sheldrick, 1996)	*h* = −17→13
*T*_min_ = 0.798, *T*_max_ = 0.970	*k* = −7→7
6237 measured reflections	*l* = −10→18

### Refinement

**Table:**

Refinement on *F*^2^	Secondary atom site location: difference Fourier map
Least-squares matrix: full	Hydrogen site location: inferred from neighbouring sites
*R*[*F*^2^ > 2σ(*F*^2^)] = 0.064	H-atom parameters constrained
*wR*(*F*^2^) = 0.139	*w* = 1/[σ^2^(*F*_o_^2^) + (0.0434*P*)^2^ + 1.1014*P*] where *P* = (*F*_o_^2^ + 2*F*_c_^2^)/3
*S* = 1.22	(Δ/σ)_max_ < 0.001
2341 reflections	Δρ_max_ = 0.35 e Å^−^^3^
154 parameters	Δρ_min_ = −0.23 e Å^−^^3^
Primary atom site location: structure-invariant direct methods	Extinction correction: none

### Special details

**Table:**

Geometry. All e.s.d.'s (except the e.s.d. in the dihedral angle between two l.s. planes) are estimated using the full covariance matrix. The cell e.s.d.'s are taken into account individually in the estimation of e.s.d.'s in distances, angles and torsion angles; correlations between e.s.d.'s in cell parameters are only used when they are defined by crystal symmetry. An approximate (isotropic) treatment of cell e.s.d.'s is used for estimating e.s.d.'s involving l.s. planes.
Refinement. Refinement of *F*^2^ against ALL reflections. The weighted *R*-factor *wR* and goodness of fit *S* are based on *F*^2^, conventional *R*-factors *R* are based on *F*, with *F* set to zero for negative *F*^2^. The threshold expression of *F*^2^ > σ(*F*^2^) is used only for calculating *R*-factors(gt) *etc*. and is not relevant to the choice of reflections for refinement. *R*-factors based on *F*^2^ are statistically about twice as large as those based on *F*, and *R*- factors based on ALL data will be even larger.

### Fractional atomic coordinates and isotropic or equivalent isotropic displacement parameters (Å^2^)

**Table:**

	*x*	*y*	*z*	*U*_iso_*/*U*_eq_	
P1	0.56727 (5)	0.36680 (12)	0.37497 (5)	0.0400 (2)	
O1	0.48666 (15)	0.5276 (3)	0.33914 (16)	0.0547 (6)	
O2	0.54859 (16)	0.1704 (3)	0.41607 (15)	0.0533 (6)	
C1	0.5943 (2)	0.3010 (5)	0.2762 (2)	0.0420 (7)	
C2	0.5828 (3)	0.4485 (6)	0.2071 (2)	0.0598 (9)	
H2A	0.5617	0.5814	0.2119	0.072*	
C3	0.6028 (3)	0.3987 (8)	0.1308 (3)	0.0797 (12)	
H3A	0.5950	0.4980	0.0843	0.096*	
C4	0.6340 (3)	0.2027 (9)	0.1236 (3)	0.0830 (13)	
H4A	0.6467	0.1694	0.0719	0.100*	
C5	0.6465 (3)	0.0569 (7)	0.1916 (3)	0.0749 (11)	
H5A	0.6685	−0.0750	0.1866	0.090*	
C6	0.6266 (2)	0.1035 (5)	0.2679 (2)	0.0564 (8)	
H6A	0.6347	0.0028	0.3139	0.068*	
C7	0.6820 (2)	0.4771 (5)	0.4685 (2)	0.0407 (7)	
C8	0.7658 (3)	0.3527 (5)	0.5140 (3)	0.0627 (9)	
H8A	0.7633	0.2162	0.4940	0.075*	
C9	0.8532 (3)	0.4287 (7)	0.5888 (3)	0.0774 (12)	
H9A	0.9091	0.3434	0.6184	0.093*	
C10	0.8579 (3)	0.6274 (7)	0.6192 (3)	0.0716 (11)	
H10A	0.9161	0.6770	0.6709	0.086*	
C11	0.7768 (3)	0.7541 (6)	0.5736 (3)	0.0703 (11)	
H11A	0.7806	0.8913	0.5932	0.084*	
C12	0.6887 (2)	0.6800 (5)	0.4982 (2)	0.0534 (8)	
H12A	0.6338	0.7677	0.4676	0.064*	
N1	0.59203 (18)	0.1330 (4)	0.61263 (18)	0.0504 (6)	
H1B	0.5862	0.1470	0.5534	0.060*	
H2B	0.5684	0.2453	0.6275	0.060*	
H3B	0.5561	0.0262	0.6140	0.060*	
H4B	0.6553	0.1138	0.6545	0.060*	
O3	0.7070 (2)	0.6051 (7)	0.7434 (2)	0.1340 (16)	
H3C	0.6462	0.5714	0.7278	0.161*	
H3D	0.7095	0.7079	0.7096	0.161*	

### Atomic displacement parameters (Å^2^)

**Table:**

	*U*^11^	*U*^22^	*U*^33^	*U*^12^	*U*^13^	*U*^23^
P1	0.0376 (4)	0.0409 (4)	0.0432 (4)	−0.0016 (3)	0.0201 (3)	−0.0034 (3)
O1	0.0422 (11)	0.0567 (13)	0.0625 (14)	0.0058 (10)	0.0216 (10)	−0.0087 (11)
O2	0.0611 (14)	0.0503 (13)	0.0534 (13)	−0.0120 (10)	0.0305 (11)	−0.0035 (10)
C1	0.0351 (14)	0.0484 (17)	0.0405 (15)	−0.0034 (13)	0.0156 (12)	−0.0034 (13)
C2	0.057 (2)	0.068 (2)	0.0519 (19)	−0.0010 (17)	0.0231 (16)	0.0048 (17)
C3	0.082 (3)	0.108 (4)	0.054 (2)	−0.006 (3)	0.035 (2)	0.014 (2)
C4	0.081 (3)	0.123 (4)	0.058 (2)	−0.008 (3)	0.042 (2)	−0.018 (3)
C5	0.072 (2)	0.084 (3)	0.078 (3)	0.008 (2)	0.043 (2)	−0.021 (2)
C6	0.0540 (19)	0.063 (2)	0.0546 (19)	0.0080 (16)	0.0267 (16)	−0.0034 (16)
C7	0.0421 (15)	0.0459 (16)	0.0368 (15)	−0.0053 (13)	0.0205 (12)	0.0007 (13)
C8	0.055 (2)	0.051 (2)	0.064 (2)	0.0025 (16)	0.0117 (17)	0.0060 (16)
C9	0.051 (2)	0.084 (3)	0.071 (2)	0.002 (2)	0.0048 (18)	0.013 (2)
C10	0.057 (2)	0.100 (3)	0.0471 (19)	−0.025 (2)	0.0145 (17)	−0.007 (2)
C11	0.076 (3)	0.071 (2)	0.067 (2)	−0.021 (2)	0.034 (2)	−0.027 (2)
C12	0.0528 (18)	0.0526 (19)	0.0547 (19)	−0.0037 (15)	0.0247 (15)	−0.0121 (15)
N1	0.0457 (14)	0.0514 (15)	0.0546 (15)	0.0003 (12)	0.0236 (12)	−0.0039 (12)
O3	0.0596 (18)	0.198 (4)	0.106 (2)	−0.038 (2)	0.0049 (17)	0.057 (3)

### Geometric parameters (Å, °)

**Table:**

P1—O1	1.495 (2)	C7—C8	1.383 (4)
P1—O2	1.503 (2)	C8—C9	1.381 (5)
P1—C1	1.804 (3)	C8—H8A	0.9300
P1—C7	1.811 (3)	C9—C10	1.358 (6)
C1—C2	1.384 (4)	C9—H9A	0.9300
C1—C6	1.392 (4)	C10—C11	1.364 (5)
C2—C3	1.384 (5)	C10—H10A	0.9300
C2—H2A	0.9300	C11—C12	1.386 (5)
C3—C4	1.372 (6)	C11—H11A	0.9300
C3—H3A	0.9300	C12—H12A	0.9300
C4—C5	1.360 (6)	N1—H1B	0.8844
C4—H4A	0.9300	N1—H2B	0.8830
C5—C6	1.378 (5)	N1—H3B	0.8823
C5—H5A	0.9300	N1—H4B	0.8782
C6—H6A	0.9300	O3—H3C	0.8585
C7—C12	1.377 (4)	O3—H3D	0.8572
			
O1—P1—O2	117.81 (13)	C12—C7—P1	122.5 (2)
O1—P1—C1	108.00 (13)	C8—C7—P1	119.2 (2)
O2—P1—C1	108.59 (13)	C9—C8—C7	120.9 (3)
O1—P1—C7	109.50 (13)	C9—C8—H8A	119.6
O2—P1—C7	106.73 (13)	C7—C8—H8A	119.6
C1—P1—C7	105.56 (13)	C10—C9—C8	120.3 (4)
C2—C1—C6	118.9 (3)	C10—C9—H9A	119.9
C2—C1—P1	119.8 (3)	C8—C9—H9A	119.9
C6—C1—P1	121.2 (2)	C9—C10—C11	119.8 (3)
C1—C2—C3	120.1 (4)	C9—C10—H10A	120.1
C1—C2—H2A	119.9	C11—C10—H10A	120.1
C3—C2—H2A	119.9	C10—C11—C12	120.5 (4)
C4—C3—C2	120.0 (4)	C10—C11—H11A	119.7
C4—C3—H3A	120.0	C12—C11—H11A	119.7
C2—C3—H3A	120.0	C7—C12—C11	120.3 (3)
C5—C4—C3	120.4 (4)	C7—C12—H12A	119.9
C5—C4—H4A	119.8	C11—C12—H12A	119.9
C3—C4—H4A	119.8	H1B—N1—H2B	109.1
C4—C5—C6	120.3 (4)	H1B—N1—H3B	109.5
C4—C5—H5A	119.8	H2B—N1—H3B	108.2
C6—C5—H5A	119.8	H1B—N1—H4B	109.7
C5—C6—C1	120.1 (4)	H2B—N1—H4B	110.6
C5—C6—H6A	119.9	H3B—N1—H4B	109.7
C1—C6—H6A	119.9	H3C—O3—H3D	111.4
C12—C7—C8	118.2 (3)		

### Hydrogen-bond geometry (Å, °)

**Table:**

*D*—H···*A*	*D*—H	H···*A*	*D*···*A*	*D*—H···*A*
N1—H1B···O2	0.88	1.94	2.814 (3)	170
N1—H2B···O1^i^	0.88	1.87	2.752 (3)	176
N1—H3B···O2^ii^	0.88	1.91	2.764 (3)	164
N1—H4B···O3^iii^	0.88	1.94	2.816 (4)	172
O3—H3C···O1^i^	0.86	1.89	2.723 (4)	164

Symmetry codes: (i) −*x*+1, −*y*+1, −*z*+1; (ii) −*x*+1, −*y*, −*z*+1; (iii) −*x*+3/2, *y*−1/2, −*z*+3/2.
